# Supplementary material for: Heritable temporal gene expression patterns correlate with metabolomic seed content in developing hexaploid oat seed
Source: Plant Biotechnol J. 2020 Jan 4;18(5):1211–22. doi: 10.1111/pbi.13286 (PMC7152608; doi:10.1111/pbi.13286)
Supplement: Supplementary file 1 — Figure S1 Transcript length distribution of the 131,457 transcripts included in the RTA. Figure S2 PCA plot of 397 samples with more than 0.5 million mapped reads based on the 500 transcripts with highest variance. Figure S3 Distribution of Pearson correlation coefficients of biological replicates from Greenhouse samples, Field samples and among samples across the two sites. Figure S4 Biological process (a), cellular compartments (b) and molecular function (c) GO terms enriched for differentially expressed transcript sets between adjacent time points. Figure S5 The 80 observed temporal transcript expression patterns identified from 25,971 differentially expressed transcripts between five pairs of adjacent time points. Figure S6 Correlation of transcript numbers (log scale) between each pair of symmetrical up‐ and down‐regulated expression patterns. Figure S7 GO categories enriched for 8 temporal transcript co‐expression sets. Figure S8 Distribution of p‐values of simple linear regression between 634 metabolites and PC1 scores of GCoE sets. Figure S9 Transcript length distribution of the 9,817 transcripts that couldn’t be aligned to the UniRef100. Figure S10 Clusters of 12 HiSeq samples based on expression profiles. Figure S11 Pearson correlation coefficients of biological replicates from 12 HiSeq samples of cv.Ogle‐C whose developing seeds were collected at 7, 14, 21, and 28 DAA. Figure S12 Heat map of genomic relationship among 22 oat lines used in this study. Table S1 A comparison of BUSCOs plant gene completeness between the RTA in this study and the first version of de novo oat seed transcriptome assembly Table S2 A list of 22 oat lines used in this study Table S3 Chi‐Square test for subcluster size distribution of the 22 temporal co‐expression sets Table S4 A list of oat transcripts homologous to biosynthetic genes of avenanthramides and fatty acids from other oat cultivars and Brachypodium distachyon Table S5 Detailed information of experimental design and 3’ [file PBI-18-1211-s002.docx]

Plant Biotechnology Journal

Supporting Information

Heritable temporal gene expression patterns correlate with metabolomic seed content in developing hexaploid oat seed

Haixiao Hu, Juan J. Gutierrez-Gonzalez, Xinfang Liu, Trevor H. Yeats, David F. Garvin, Owen A. Hoekenga, Mark E. Sorrells, Michael A. Gore, Jean-Luc Jannink

**
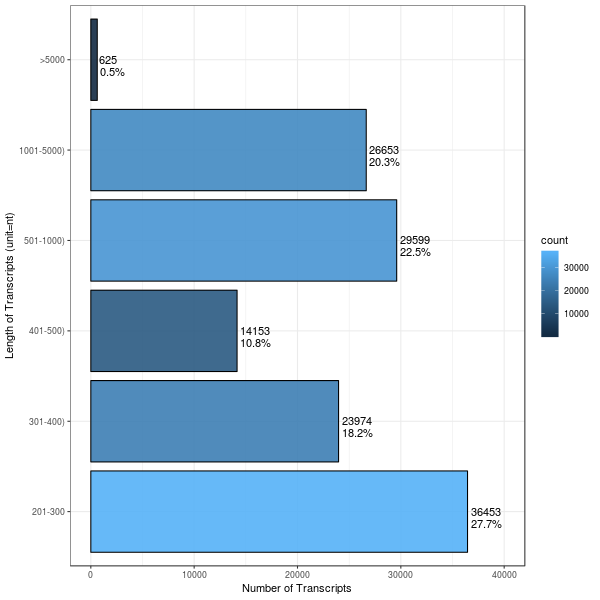
**

**Figure S1** Transcript length distribution of the 131,457 transcripts included in the RTA

**
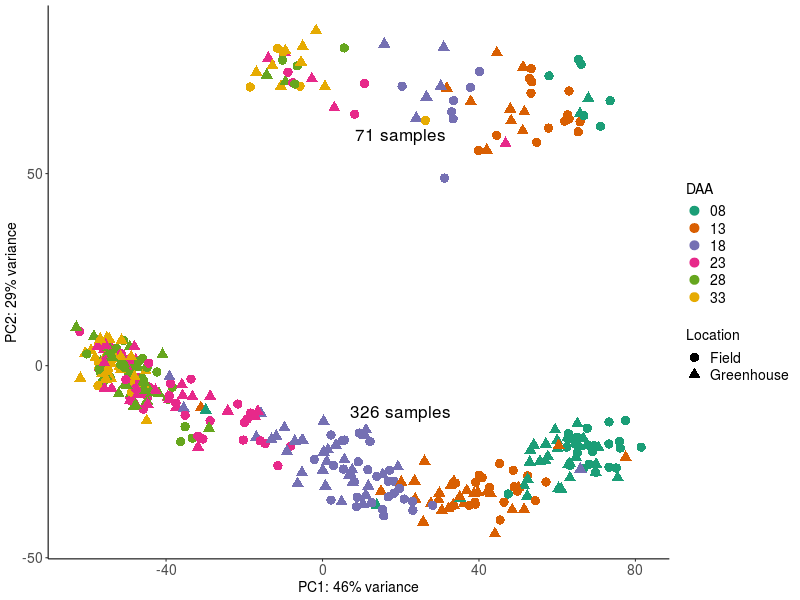
**

**Figure S2** PCA plot of 397 samples with more than 0.5 million mapped reads based on the 500 transcripts with highest variance.

**
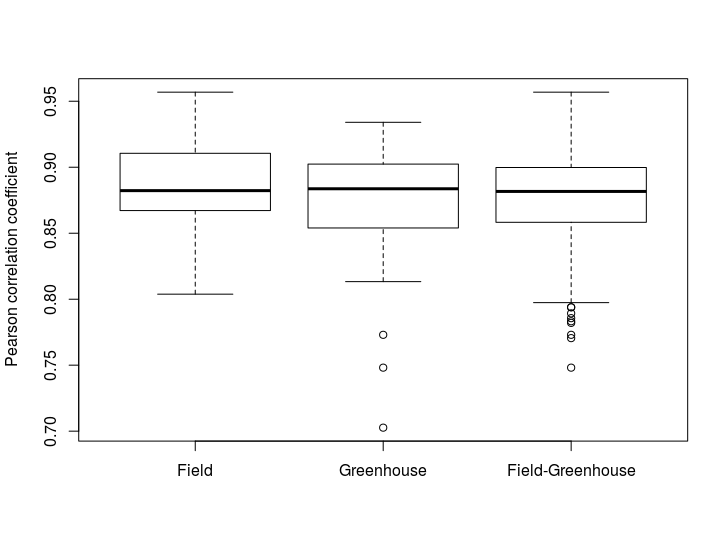
**

**Figure S3** Distribution of Pearson correlation coefficients of biological replicates from Greenhouse samples, Field samples and among samples across the two sites

**
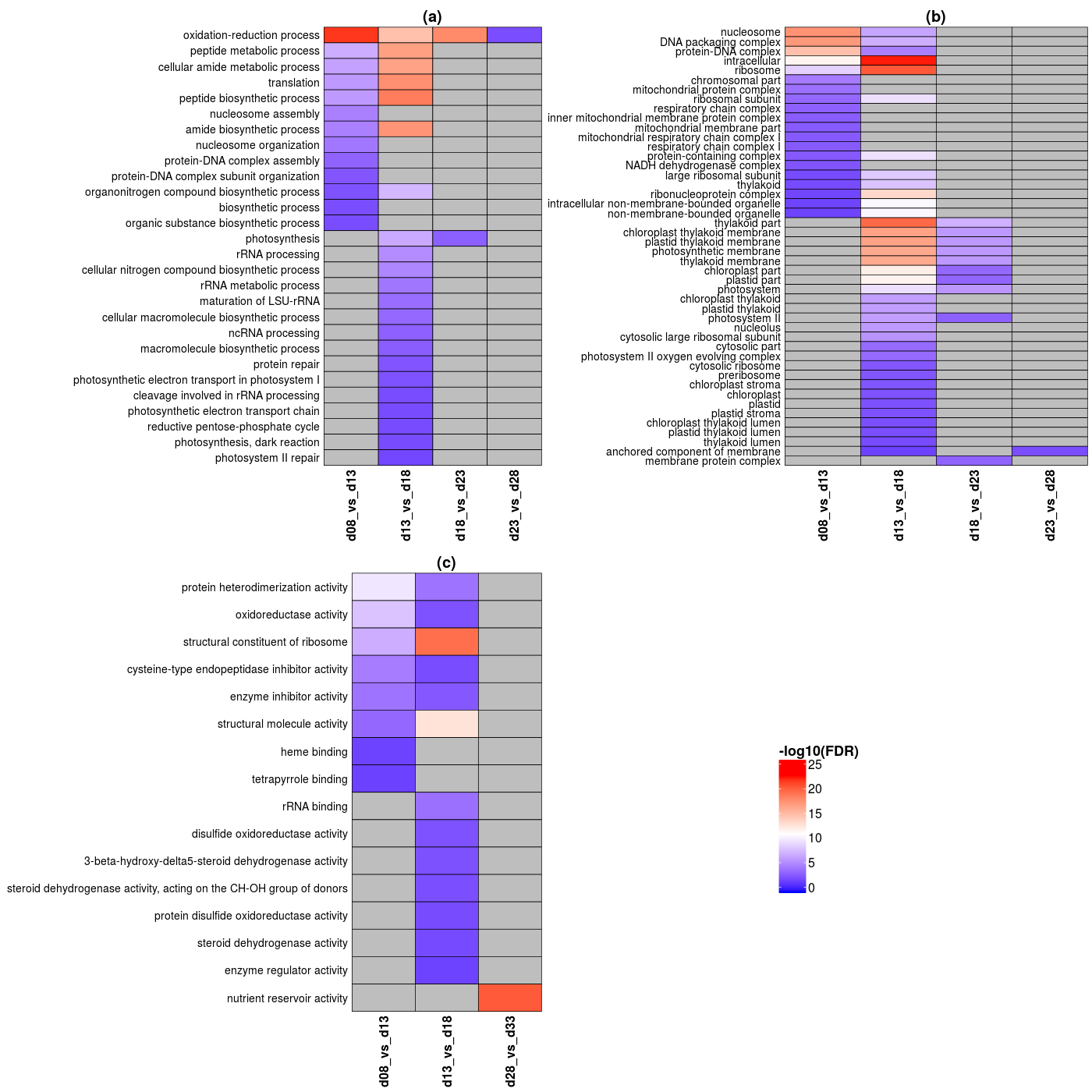
**

**Figure S4** Biological process (a), cellular compartments (b) and molecular function (c) GO terms enriched for differentially expressed transcript sets between adjacent time points. FDR adjusted p-values < 0.01 (in -log10 scale) were colored between blue and red, and cells without GO terms assigned were colored in gray.

**
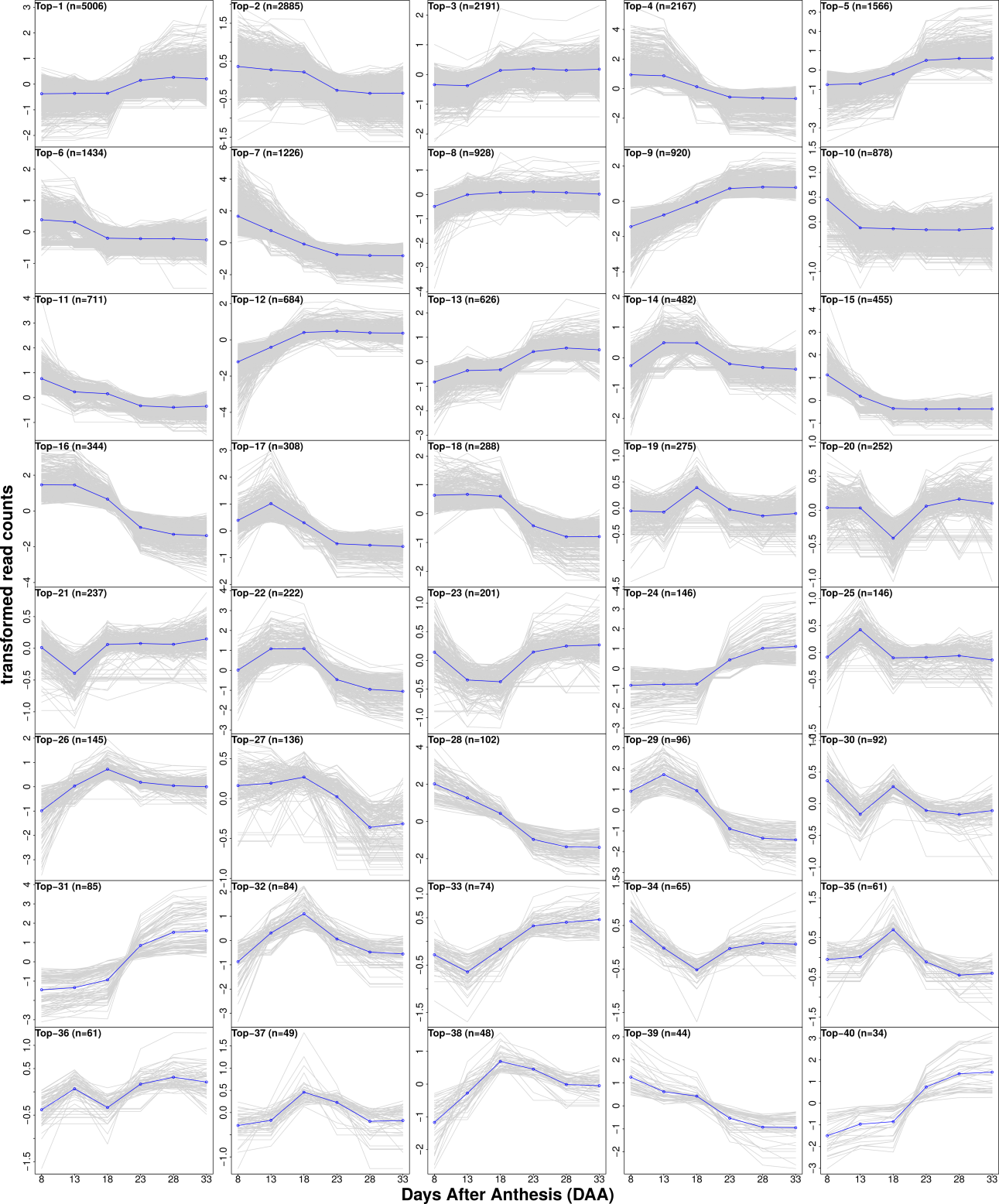
**

**Figure S5** Top 1-40 of the 80 observed temporal transcript expression patterns identified from 25,971 differentially expressed transcripts between five pairs of adjacent time points


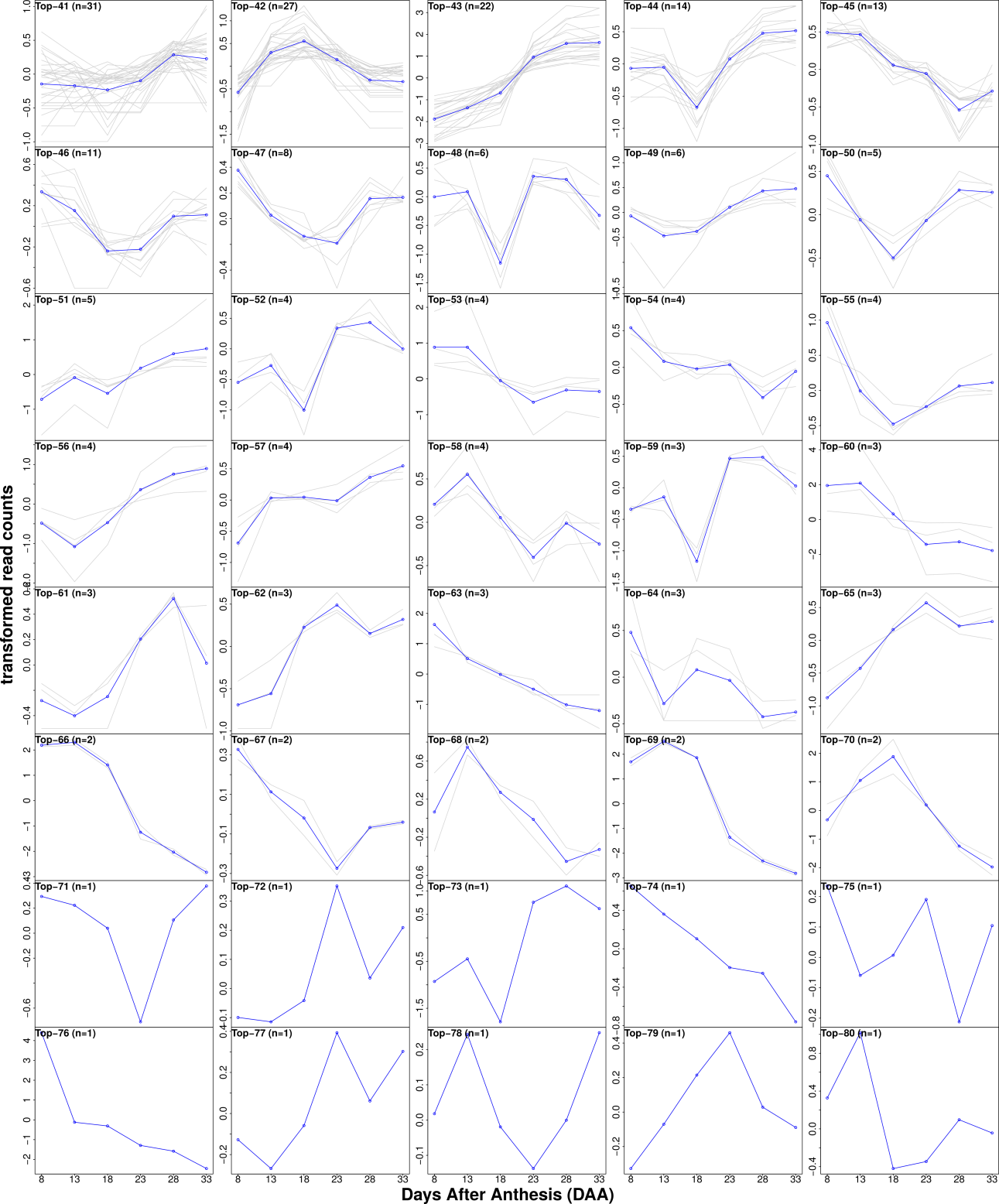


**Figure S5 (continued)** Top 41-80 of the 80 observed temporal transcript expression patterns identified from 25,971 differentially expressed transcripts between five pairs of adjacent time points


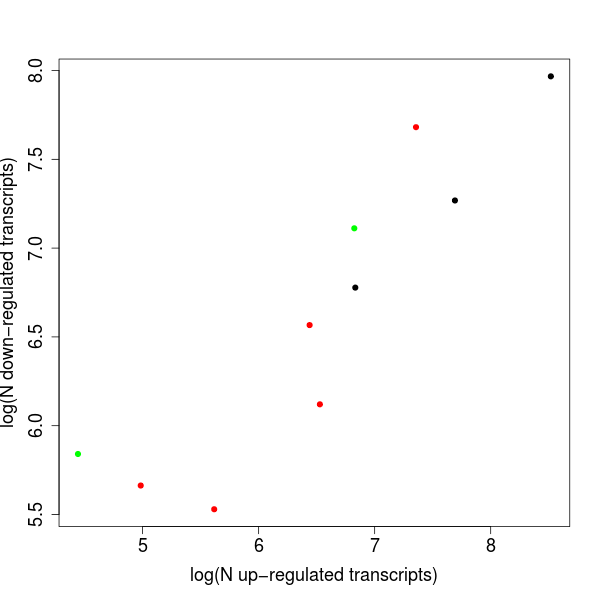


**Figure S6** Correlation of transcript numbers (log scale) between each pair of symmetrical up- and down-regulated expression patterns. Each point represents a pair of symmetrical up- and down-regulated expression patterns. The number of transcripts in the up-regulated pattern on the x-axis and the number of transcript in the down-regulated pattern on the y-axis. Black points have one differential expression event, red points two, and green points three such events.

**
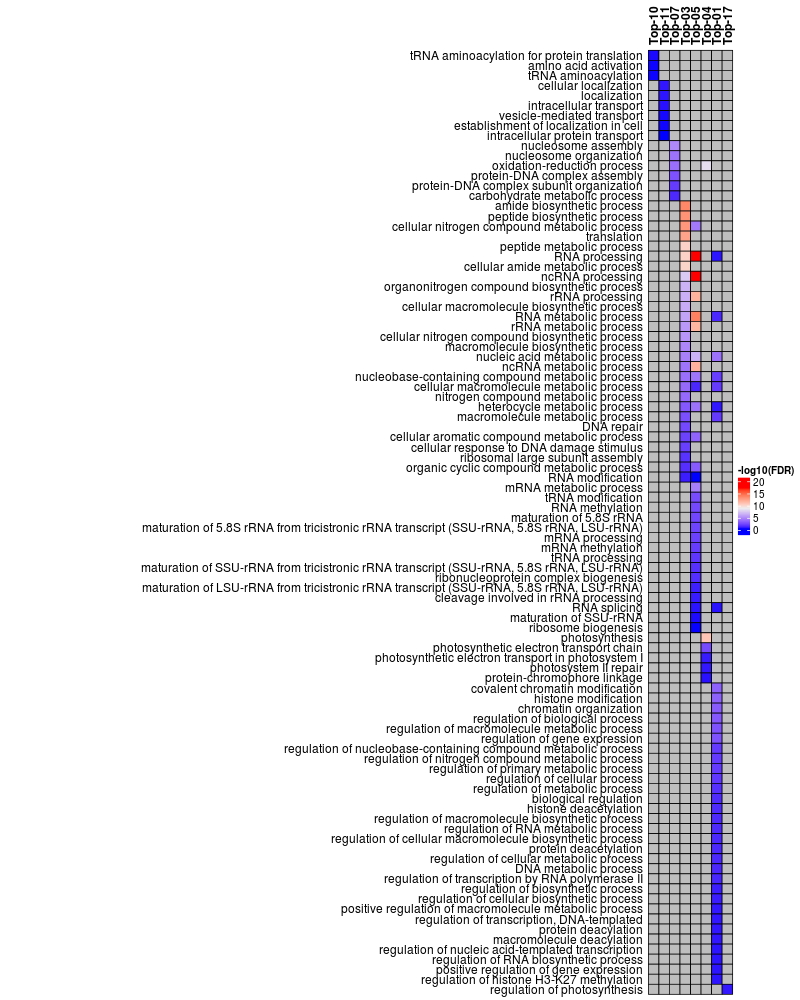
**

**Figure S7** GO categories enriched for 8 temporal transcript co-expression sets. FDR adjusted p-values < 0.01 (in -log10 scale) were colored between blue and red, and cells without GO terms assigned were colored in gray.


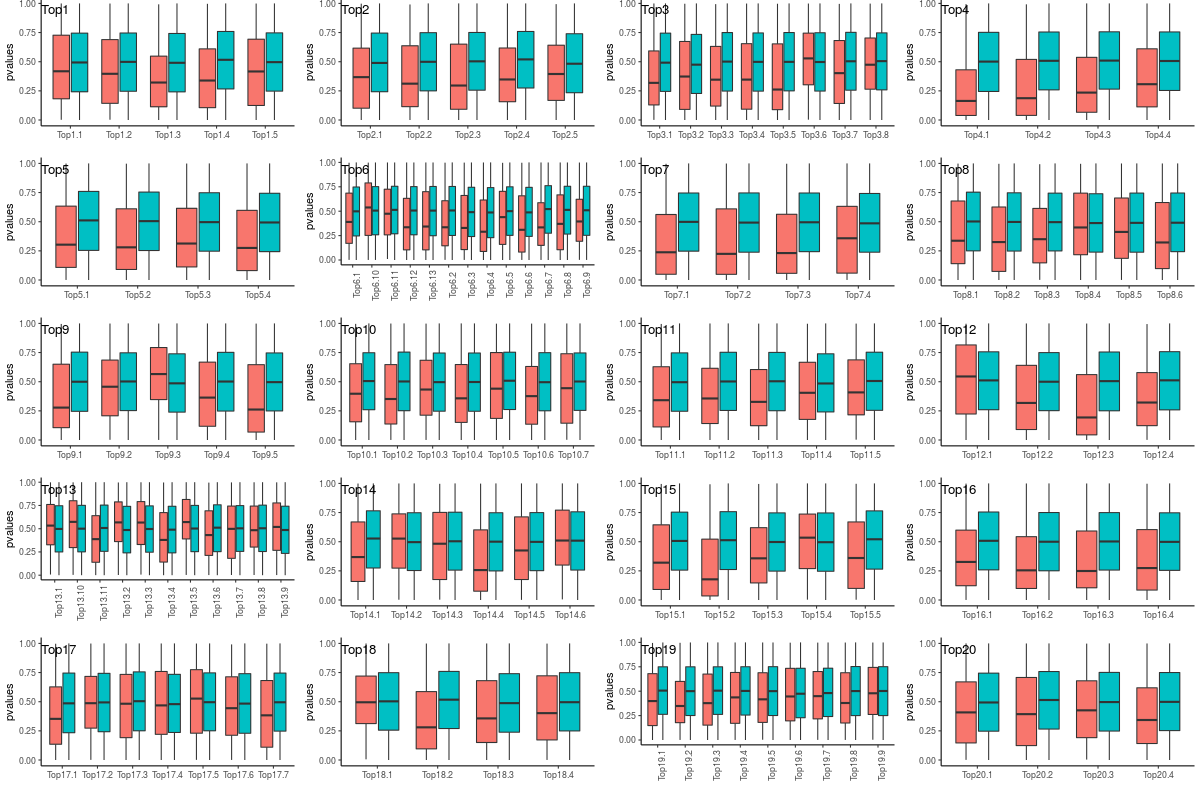


**Figure S8** Distribution of p-values of simple linear regression between 634 metabolites and PC1 scores of GCoE sets. Red boxes contained p-values from real data, and blue boxes contained p-values from 100 permutations.

**
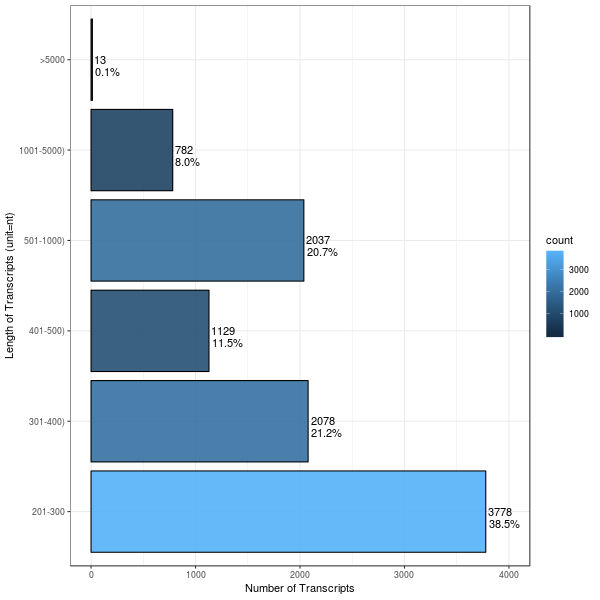
**

**Figure S9** Transcript length distribution of the 9,817 transcripts that couldn’t be aligned to the UniRef100


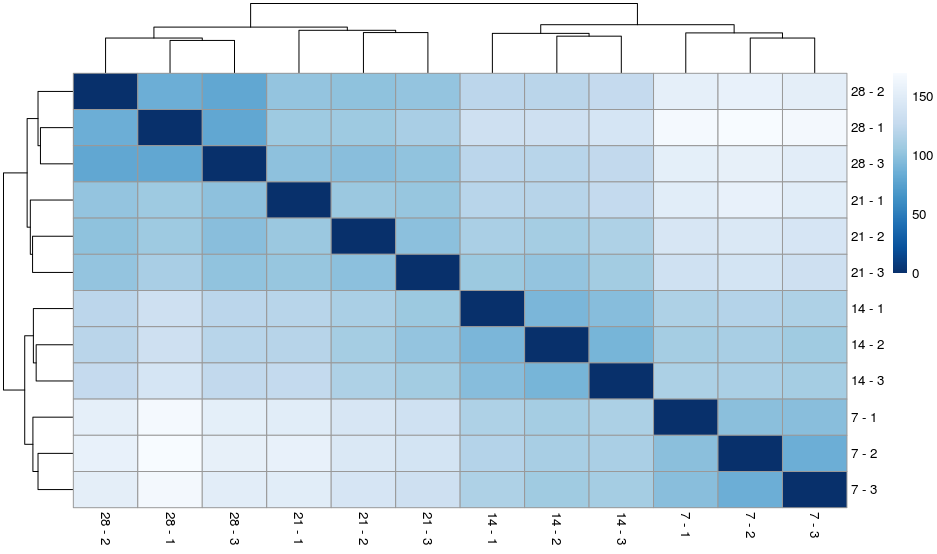


**Figure S10** clusters of 12 HiSeq samples based on expression profiles. Euclidean distances between samples were colored between dark blue and light blue.

**
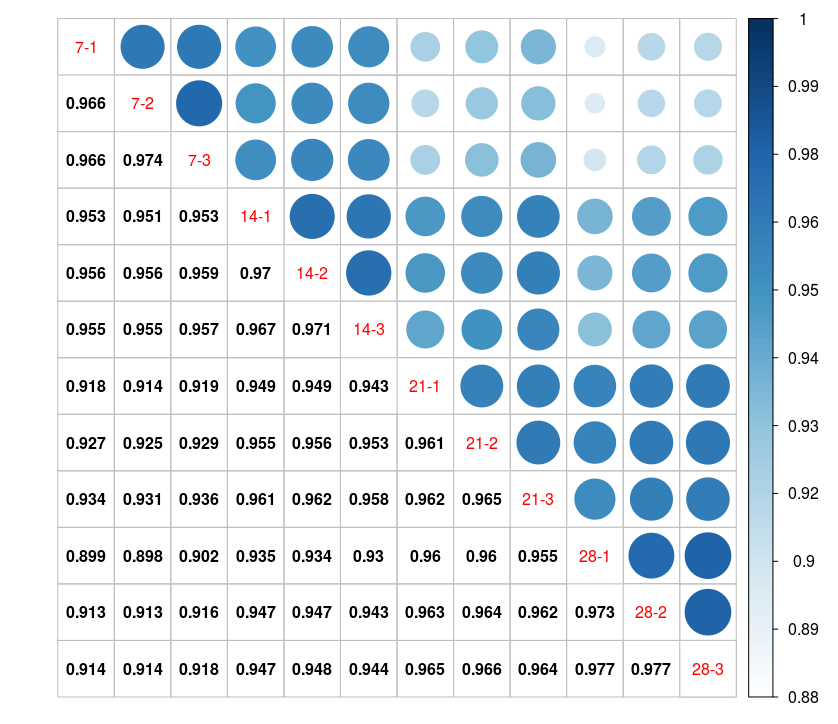
**

**Figure S11** Pearson correlation coefficients of biological replicates from 12 HiSeq samples of cv.Ogle-C whose developing seeds were collected at 7, 14, 21, and 28 DAA

**
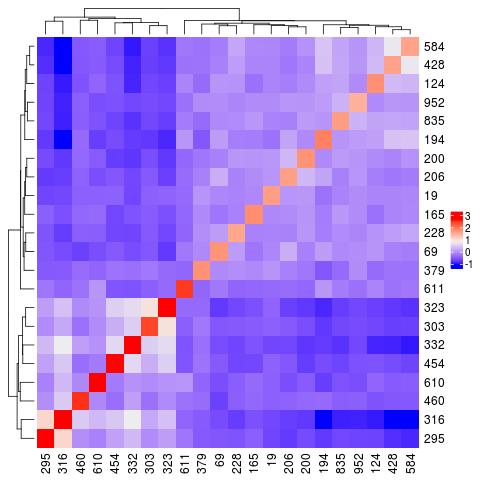
**

**Figure S12** Heatmap of genomic relationship among 22 oat lines used in this study

**Table S1** A comparison of BUSCOs plant gene completeness between the RTA in this study and the first version of *de novo* oat seed transcriptome assembly (*dn*OST, Gutierrez-Gonzalez et al. 2013)

| BUSCO Statistics | the RTA | *dn*OST |
| --- | --- | --- |
| Complete BUSCOs | 1212 (84.17%) | 412 (28.61%) |
| Complete and single-copy BUSCOs | 1188 (82.50%) | 116 (8.06%) |
| Complete and duplicated BUSCOs | 24 (1.67%) | 296 (20.56%) |
| Fragmented BUSCOs | 148 (10.28%) | 201 (13.96%) |
| Missing BUSCOs | 80 (5.56%) | 827 (57.43%) |
| Total BUSCO groups searched | 1440 | 1440 |

**Table S2** A list of 22 oat lines used in this study

| GID | T3Oat_Name^1^ |
| --- | --- |
| 19 | IL05-8515 |
| 69 | X8826-1 |
| 124 | SHERWOOD |
| 165 | MN08130 |
| 194 | ANDREW |
| 200 | EXCEL |
| 206 | WOODBURN |
| 228 | 95AB12770 |
| 295 | TIFT |
| 303 | FL0238BSB-22 |
| 316 | TX02U7479 |
| 323 | TX07CS1402 |
| 332 | HORIZON270 |
| 428 | PI344841 |
| 454 | PERDEBERG |
| 460 | CIAV5389 |
| 584 | ONOHOJSKIJ_A-547 |
| 610 | QUALITY\|PI289587 |
| 611 | CIAV6218 |
| 835 | IA111003 |
| 952 | M2-2 |
| 379 | CORRAL |

^1^T3Oat_Name= line names were consistent with that on T3/Oat (https://triticeaetoolbox.org/oat/)

**Table S3** Chi-Square test for sub-cluster size distribution of the 22 temporal co-expression sets. At significant level of 0.05, the critical p-value after Bonferroni correction is 0.05/22=0.00227.

| Temporal co-expression set* | P-value |
| --- | --- |
| Top-8 | 0.994548451 |
| Top-10 | 0.999286221 |
| Top-12 | 0.497109215 |
| Top-15 | 0.003939038 |
| Top-13 | 0.108831006 |
| Top-11 | 0.344207141 |
| Top-9 | 0.380203442 |
| Top-7 | 0.608797221 |
| Top-3 | 0.895528366 |
| Top-6 | 0.419936316 |
| Top-5 | 0.273601551 |
| Top-4 | 0.594794097 |
| Top-31 | 0.021221027 |
| Top-16 | 0.133281338 |
| Top-1 | 0.637929437 |
| Top-2 | 0.83840654 |
| Top-24 | 0.744979403 |
| Top-18 | 0.210760149 |
| Top-19 | 0.980442768 |
| Top-20 | 0.35296887 |
| Top-14 | 0.962213032 |
| Top-17 | 0.549011073 |

*The temporal co-expression sets were ordered the same as in Figure 4.

**Table S4** A list of oat transcripts homologous to biosynthetic genes of avenanthremides and fatty acids from other oat cultivars and *Brachypodium distachyon*

| **Genename** | **Source** | **Transcript name** | **reference** | **query_len^a^** | **ref_len^b^** | **aln_length^c^** | **pct_ident^d^** |
| --- | --- | --- | --- | --- | --- | --- | --- |
| **Avenanthramides biosynthetic genes** | | | | | | | |
| CCoA3H | Arabidopsis | TRINITY_DN6473_c0_g1_i1 | NM_180006.2 | 1942 | 1885 | 1362 | 67.621 |
| CCoA3H | Brachypodium | TRINITY_DN6473_c0_g1_i1 | KQK05634 | 1942 | 2031 | 1954 | 86.131 |
| CCoAOMT | Oat | TRINITY_DN3508_c0_g1_i7 | AB076979.1 | 1238 | 622 | 604 | 99.834 |
| HHT1 | Oat | TRINITY_DN5172_c0_g1_i4 | AB076980.1 | 1598 | 1755 | 1343 | 79.449 |
| HHT2 | Oat | TRINITY_DN5172_c0_g1_i4 | AB076981.1 | 1598 | 1770 | 1343 | 78.555 |
| HHT3 | Oat | TRINITY_DN5172_c0_g1_i4 | AB076982.1 | 1598 | 1659 | 1343 | 79.598 |
| HHT4 | Oat | TRINITY_DN5172_c0_g1_i4 | AB076983.1 | 1598 | 1100 | 1088 | 97.151 |
| **Fatty Acids biosynthetic genes** | | | | | | | |
| ACCase | Brachypodium | TRINITY_DN146_c0_g1_i8 | BRADI_5g03860 | 7812 | 8783 | 7598 | 88.694 |
| DGAT1/TAG1 | Brachypodium | TRINITY_DN151_c0_g1_i4 | BRADI_1g37750 | 2878 | 3009 | 925 | 72.757 |
| FAB1/KAS2 | Brachypodium | TRINITY_DN207_c0_g1_i1 | BRADI_1g60300 | 2876 | 2498 | 1851 | 87.088 |
| FAB2 | Brachypodium | TRINITY_DN2740_c0_g1_i5 | BRADI_2g58930 | 5078 | 1792 | 1610 | 86.957 |
| FAD2 | Brachypodium | TRINITY_DN268_c0_g1_i4 | BRADI_3g53370 | 1883 | 2351 | 1665 | 85.526 |
| FAD3 | Brachypodium | TRINITY_DN10818_c0_g1_i3 | BRADI_1g65580 | 1198 | 2817 | 1195 | 82.343 |
| FAE1/KCS18 | Brachypodium | TRINITY_DN1011_c0_g1_i5 | BRADI_2g16050 | 1990 | 2481 | 1447 | 81.064 |
| FATB | Brachypodium | TRINITY_DN2629_c0_g1_i1 | BRADI_1g51170 | 2394 | 4407 | 1368 | 88.085 |
| GPAT9 | Brachypodium | TRINITY_DN5048_c0_g1_i7 | BRADI_1g25790 | 1490 | 1540 | 1460 | 86.37 |
| LPCAT1 | Brachypodium | TRINITY_DN3352_c0_g1_i1 | BRADI_3g51577 | 1846 | 1671 | 1634 | 88.433 |
| PAH1 | Brachypodium | TRINITY_DN1042_c0_g1_i6 | BRADI_2g23040 | 4252 | 4162 | 2485 | 82.817 |
| PDAT1 | Brachypodium | TRINITY_DN2921_c0_g1_i1 | BRADI_4g31540 | 2494 | 2601 | 2531 | 86.448 |
| WRI1 | Brachypodium | TRINITY_DN331_c0_g1_i1 | BRADI_4g43877 | 3900 | 2035 | 1223 | 80.376 |

^a^query_len= query sequence length;

^b^ref_len = reference sequence length;

^c^aln_length= alignment length;

^d^pct_ident= percent identity

**Table S5** Detailed information of experimental design and 3’ RNASeq sample names

| **Location** | **Block** | **Plot** | **GID** | **T3Oat_Name^1^** | **RNASeq_Samplename^2^** |
| --- | --- | --- | --- | --- | --- |
| Greenhouse | 1 | 1 | 206 | WOODBURN | G001 |
| Greenhouse | 1 | 2 | 69 | X8826-1 | G002 |
| Greenhouse | 1 | 3 | 124 | SHERWOOD | G003 |
| Greenhouse | 1 | 4 | 316 | TX02U7479 | G004 |
| Greenhouse | 1 | 5 | 19 | IL05-8515 | G005 |
| Greenhouse | 1 | 7 | 332 | HORIZON270 | G007 |
| Greenhouse | 1 | 8 | 835 | IA111003 | G008 |
| Greenhouse | 1 | 9 | 428 | PI344841 | G009 |
| Greenhouse | 1 | 10 | 379 | CORRAL | G010 |
| Greenhouse | 1 | 11 | 165 | MN08130 | G011 |
| Greenhouse | 1 | 13 | 228 | 95AB12770 | G013 |
| Greenhouse | 1 | 14 | 610 | QUALITY\|PI289587 | G014 |
| Greenhouse | 1 | 15 | 952 | M2-2 | G015 |
| Greenhouse | 1 | 16 | 460 | CIAV5389 | G016 |
| Greenhouse | 1 | 17 | 323 | TX07CS1402 | G017 |
| Greenhouse | 1 | 18 | 454 | PERDEBERG | G018 |
| Greenhouse | 1 | 19 | 200 | EXCEL | G019 |
| Greenhouse | 1 | 20 | 303 | FL0238BSB-22 | G020 |
| Greenhouse | 1 | 21 | 584 | ONOHOJSKIJ_A-547 | G021 |
| Greenhouse | 1 | 22 | 194 | ANDREW | G022 |
| Greenhouse | 1 | 24 | 611 | CIAV6218 | G024 |
| Greenhouse | 1 | 25 | 295 | TIFT | G025 |
| Greenhouse | 2 | 26 | 19 | IL05-8515 | G026 |
| Greenhouse | 2 | 28 | 124 | SHERWOOD | G028 |
| Greenhouse | 2 | 29 | 332 | HORIZON270 | G029 |
| Greenhouse | 2 | 30 | 206 | WOODBURN | G030 |
| Greenhouse | 2 | 31 | 584 | ONOHOJSKIJ_A-547 | G031 |
| Greenhouse | 2 | 32 | 303 | FL0238BSB-22 | G032 |
| Greenhouse | 2 | 33 | 428 | PI344841 | G033 |
| Greenhouse | 2 | 34 | 611 | CIAV6218 | G034 |
| Greenhouse | 2 | 35 | 460 | CIAV5389 | G035 |
| Greenhouse | 2 | 36 | 228 | 95AB12770 | G036 |
| Greenhouse | 2 | 37 | 454 | PERDEBERG | G037 |
| Greenhouse | 2 | 38 | 69 | X8826-1 | G038 |
| Greenhouse | 2 | 39 | 835 | IA111003 | G039 |
| Greenhouse | 2 | 40 | 610 | QUALITY\|PI289587 | G040 |
| Greenhouse | 2 | 41 | 323 | TX07CS1402 | G041 |
| **Location** | **Block** | **Plot** | **GID** | **T3Oat_Name^1^** | **RNASeq_Samplename^2^** |
| Greenhouse | 2 | 42 | 952 | M2-2 | G042 |
| Greenhouse | 2 | 43 | 165 | MN08130 | G043 |
| Greenhouse | 2 | 45 | 295 | TIFT | G045 |
| Greenhouse | 2 | 47 | 194 | ANDREW | G047 |
| Greenhouse | 2 | 48 | 379 | CORRAL | G048 |
| Greenhouse | 2 | 49 | 316 | TX02U7479 | G049 |
| Greenhouse | 2 | 50 | 200 | EXCEL | G050 |
| Field | 1 | 803 | 124 | SHERWOOD | C803 |
| Field | 1 | 804 | 200 | EXCEL | C804 |
| Field | 1 | 806 | 303 | FL0238BSB-22 | C806 |
| Field | 1 | 807 | 316 | TX02U7479 | C807 |
| Field | 1 | 808 | 428 | PI344841 | C808 |
| Field | 1 | 809 | 206 | WOODBURN | C809 |
| Field | 1 | 810 | 295 | TIFT | C810 |
| Field | 1 | 811 | 19 | IL05-8515 | C811 |
| Field | 1 | 812 | 228 | 95AB12770 | C812 |
| Field | 1 | 813 | 379 | CORRAL | C813 |
| Field | 1 | 814 | 952 | M2-2 | C814 |
| Field | 1 | 815 | 194 | ANDREW | C815 |
| Field | 1 | 816 | 454 | PERDEBERG | C816 |
| Field | 1 | 817 | 165 | MN08130 | C817 |
| Field | 1 | 818 | 69 | X8826-1 | C818 |
| Field | 1 | 819 | 835 | IA111003 | C819 |
| Field | 1 | 820 | 610 | QUALITY\|PI289587 | C820 |
| Field | 1 | 821 | 323 | TX07CS1402 | C821 |
| Field | 1 | 822 | 584 | ONOHOJSKIJ_A-547 | C822 |
| Field | 1 | 823 | 332 | HORIZON270 | C823 |
| Field | 1 | 824 | 611 | CIAV6218 | C824 |
| Field | 1 | 825 | 460 | CIAV5389 | C825 |
| Field | 2 | 826 | 200 | EXCEL | C826 |
| Field | 2 | 827 | 228 | 95AB12770 | C827 |
| Field | 2 | 828 | 611 | CIAV6218 | C828 |
| Field | 2 | 829 | 295 | TIFT | C829 |
| Field | 2 | 830 | 19 | IL05-8515 | C830 |
| Field | 2 | 831 | 454 | PERDEBERG | C831 |
| Field | 2 | 832 | 194 | ANDREW | C832 |
| Field | 2 | 834 | 316 | TX02U7479 | C834 |
| Field | 2 | 835 | 835 | IA111003 | C835 |
| Field | 2 | 836 | 206 | WOODBURN | C836 |
| **Location** | **Block** | **Plot** | **GID** | **T3Oat_Name^1^** | **RNASeq_Samplename^2^** |
| Field | 2 | 837 | 610 | QUALITY\|PI289587 | C837 |
| Field | 2 | 838 | 952 | M2-2 | C838 |
| Field | 2 | 839 | 584 | ONOHOJSKIJ_A-547 | C839 |
| Field | 2 | 840 | 165 | MN08130 | C840 |
| Field | 2 | 841 | 332 | HORIZON270 | C841 |
| Field | 2 | 842 | 428 | PI344841 | C842 |
| Field | 2 | 845 | 69 | X8826-1 | C845 |
| Field | 2 | 846 | 124 | SHERWOOD | C846 |
| Field | 2 | 847 | 303 | FL0238BSB-22 | C847 |
| Field | 2 | 848 | 323 | TX07CS1402 | C848 |
| Field | 2 | 849 | 379 | CORRAL | C849 |
| Field | 2 | 850 | 460 | CIAV5389 | C850 |

^1^T3Oat_Name= line names were consistent with that on T3/Oat (https://triticeaetoolbox.org/oat/)

^2^RNASeq_Samplename= run name of 3’ RNASeq samples

**Appendix S1** A fasta file containing the 131,457 transcript sequences of the RTA.

**Appendix S2** An expression matrix of 59,815 transcripts by 397 samples. Sample names were coded as combination of Location, GID, DAA and block ID, which were described in Table S5. Expression abundances were normalized by sample-specific size factor and then variance stabilization transformed using DESseq2.

**Appendix S3** Lists of differentially expressed genes between each pair of time points.

**Appendix S4** A file containing *de novo* assembled RTA transcripts annotation.

**Appendix S5** raw reads of the 12 libraries sequenced in paired-end mode with 100 cycles on the Illumina HiSeq 2000 platform.

**Appendix S6** raw reads of one library constructed from a pool of RNA from four developmental stages and sequenced by the Illumina MiSeq platform.

**Appendix S7** raw reads of 419 3’ RNASeq libraries sequenced by NextSeq500/HiSeq2000 with a 150 nt single-end run.

Data sets of appendices S1 to S7 are available on the CyVerse Data Commons. DOI: 10.25739/7y0n-de49 (Hu 2019). CyVerse Data Store file path:

<http://datacommons.cyverse.org/browse/iplant/home/shared/commons_repo/curated/HaixiaoHu_PBJOatTranscriptome_Oct2019>.

References

Haixiao, H. (2019) Heritable temporal gene expression patterns correlate with metabolomic seed content in developing hexaploid oat seed – Supporting information. CyVerse Data Commons. https://doi.org/10.25739/7y0n-de49
